# Relationship Between Weather Conditions and Risk Factors for Cerebral Aneurysm Rupture in the Development of Subarachnoid Hemorrhage

**DOI:** 10.7759/cureus.86097

**Published:** 2025-06-15

**Authors:** Yuki Sakaeyama, Yutaka Fuchinoue, Masaaki Nemoto, Ryo Matsuzaki, Shuhei Kubota, Mitsuyoshi Abe, Sayaka Terazono, Nobuo Sugo

**Affiliations:** 1 Neurosurgery, Toho University, Tokyo, JPN; 2 Neurosurgery (Sakura), Toho University, Chiba, JPN

**Keywords:** aneurysmal subarachnoid hemorrhage, meteorology, ruptured cerebral aneurysm, temperature, weather

## Abstract

Introduction

Seasonal variations have been proposed as potential contributors to the risk of cerebral aneurysm rupture. The Unruptured Cerebral Aneurysm Study of Japan (UCAS Japan) score is a validated tool to assess the risk of aneurysm rupture, incorporating six factors: age, sex, hypertension, aneurysm size, location, and the presence of a daughter sac. Risk stratification is as follows: 0-3 (Risk I; 3-year rupture rate, 0.2%-0.9%), 4-5 (Risk II; 1.4%-2.3%), 6-8 (Risk III; 3.7%-7.6%), and ≥9 (Risk IV; ≥17%). This study investigates the association between meteorological conditions and the occurrence of subarachnoid hemorrhage (SAH) due to aneurysm rupture, with patients' risk levels categorized according to their UCAS Japan scores.

Methods

This study included 137 patients diagnosed with SAH who were admitted to our hospital between January 2014 and December 2023. Meteorological variables, including temperature, atmospheric pressure, and precipitation, were analyzed to examine their association with the onset of SAH.

Results

The temperature difference between one day before onset and the day of onset was significantly greater in Group IV compared to Group II (p< 0.05). Additionally, the temperature differences between the day of onset and 1, 2, and 3 days before onset were significantly larger in Group IV than in Group III (p< 0.01, p < 0.01, and p < 0.05, respectively). The temperature difference between the day of onset and 1, 2, and 3 days before onset was significantly larger in Group IV than in Groups I, II, and III (p < 0.01). No significant differences were observed among the groups in terms of atmospheric pressure, precipitation, or seasonal variation.

Conclusion

Temperature drops are associated with SAH onset in patients with high-risk aneurysms. This study highlights the importance of considering weather-related factors, particularly temperature fluctuations, when assessing the risk of aneurysm rupture. Further research is warranted to validate these findings.

## Introduction

Subarachnoid hemorrhage (SAH) resulting from a ruptured cerebral aneurysm is a life-threatening condition with high mortality and limited treatment options [1]. Seasonal variations have been proposed as contributing factors to aneurysm rupture [2], prompting ongoing discussion about whether specific weather conditions may increase the risk of SAH. Several studies have suggested associations with lower temperatures [3-6], fluctuations in atmospheric pressure [7,8], precipitation, reduced sunlight exposure, and lower relative humidity [9]. In contrast, other studies have reported little correlation between weather conditions and the onset of SAH [10,11], leaving the relationship between SAH and meteorological factors unresolved. In addition to environmental influences, established risk factors for aneurysm rupture include patient-specific characteristics and lifestyle factors such as hypertension, smoking, and excessive alcohol consumption [12,13]. The Unruptured Cerebral Aneurysm Study in Japan (UCAS Japan), a large-scale prospective cohort study, followed 6,697 cerebral aneurysms in 5,720 patients over three years and reported an annual rupture rate of 0.95% [14]. This study identified aneurysm size, location, and the presence of a daughter sac as key predictors of rupture risk [14]. Furthermore, based on data from the UCAS Japan, Tominari et al. developed the UCAS Japan score, which incorporates six variables (age, sex, hypertension, cerebral aneurysm size, location of the aneurysm, and presence of a daughter sac), to stratify rupture risk into four categories over a three-year period [15]. This scoring system provides a comprehensive assessment that reflects both patient-related and aneurysm-specific risk factors. In this study, we utilized the UCAS Japan score to evaluate cases of SAH caused by ruptured cerebral aneurysms.

This study aimed to investigate whether the influence of weather conditions on SAH onset varies according to the stratified risk of aneurysm rupture as defined by the UCAS Japan score.

## Materials and methods

Patient population

**Figure 1:**
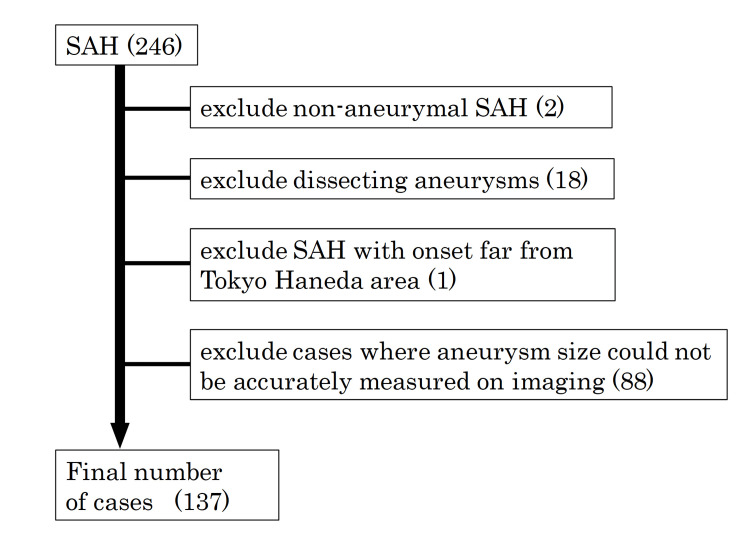
Patient selection Numbers in parentheses indicate the number of cases. SAH: subarachnoid hemorrhage.

This study included 246 patients with SAH who were admitted to Toho University Medical Center Omori Hospital between January 2014 and December 2023. Patients were excluded if they had non-aneurysmal SAH, dissecting vertebral artery aneurysms, or if the SAH occurred outside Tokyo’s 23 wards, as these locations were considered geographically distant from our hospital (Figure 1). Additionally, cases in which aneurysm size could not be accurately determined through imaging were excluded. After applying these criteria, a total of 137 patients were included in the final analysis.

Data management

Age, sex, hypertension, diabetes mellitus, dyslipidemia, smoking, alcohol consumption, use of antiplatelet agents and/or anticoagulants, and location of cerebral aneurysms were retrospectively investigated. In this study, the date of onset of SAH was defined as the date of headache onset, rather than the date of hospital admission. On the day of onset, aneurysm characteristics such as the size (mm), major axis (mm), width (mm), depth (mm), and neck diameter (mm) were measured using either three-dimensional computed tomography angiography (3DCTA) or digital subtraction angiography. From these values, the aneurysm volume (mL) was calculated using the formula: 4/3π × major axis (mm) × width (mm) × depth (mm). The dome-to-neck ratio was also determined. In addition, the presence of multiple aneurysms and daughter sacs was assessed. All aneurysm measurements were obtained under standardized imaging conditions, including consistent window width and level settings. Image interpretation was independently performed by at least two experienced board-certified neurosurgeons from the Japan Neurosurgical Society, and any discrepancies were resolved through consensus.

The UCAS Japan score

Using these data, 137 patients with SAH were categorized into four risk groups according to their UCAS Japan scores. The UCAS Japan score was calculated based on the following: age (<70 years: 0; ≥70 years: 1), sex (male: 0; female: 1), presence of hypertension (absent: 0; present: 1), aneurysm size (3 to <7 mm: 0; 7 to <10 mm: 2; 10 to <20 mm: 5; ≥20 mm: 8), aneurysm location (internal carotid artery, not internal carotid posterior communicating artery aneurysm (ICPC): 0; anterior cerebral artery or vertebral artery: 1; middle cerebral artery or basilar artery: 2; anterior communicating artery or ICPC: 3), and presence of a daughter sac (absent: 0; present: 1) [15]. Total scores were then classified into four risk levels: 0-3 points (Risk I; 3-year rupture rate: 0.2%-0.9%), 4-5 points (Risk II; 1.4%-2.3%), 6-8 points (Risk III; 3.7%-7.6%), and ≥9 points (Risk IV; ≥17%) [15]. In this study, cerebral aneurysm size was defined as the maximum value among the major, transverse, and depth axes. Aneurysms with a diameter less than 3 mm were assigned a size score of “0” in the UCAS Japan scoring system.

Weather data

Weather data were obtained from the Japan Meteorological Agency website [16]. Our hospital is situated approximately 5.5 km from the Tokyo Aviation Weather Service Center at Haneda Airport and about 13 km from the Tokyo Regional Headquarters in Kitanomaru Park, Chiyoda Ward, Tokyo. Data on daily average temperature (°C), maximum temperature (°C), minimum temperature (°C), and daily precipitation (mm) were collected from the Tokyo Aviation Weather Service Center, while data on average atmospheric pressure (hPa) were obtained from the Tokyo Regional Headquarters. From these records, the difference between the maximum and minimum temperatures on the day of onset was calculated. Furthermore, the differences between the average temperature on the day of SAH onset and the average temperatures 1, 2, 3, 4, and 5 days prior were computed. Similarly, changes in atmospheric pressure between the day of onset and 1, 2, 3, 4, and 5 days before onset were assessed. Seasons were categorized as follows: spring (March-May), summer (June-August), autumn (September-November), and winter (December-February).

Statistical analysis

Statistical analysis was performed using the Kruskal-Wallis test to compare differences among the four UCAS Japan score groups. For pairwise comparisons between groups, the Steel-Dwass multiple comparison test was employed. A p-value of less than 0.05 was considered statistically significant. This study was approved by the Ethics Committee of Toho University School of Medicine (Approval No. A24003). Informed consent was obtained from all patients and participants on an opt-out basis.

## Results

Patient characteristics and the UCAS Japan score

The 137 patients with SAH were stratified into UCAS Japan score grades I-IV and compared based on patient characteristics (Table 1). Statistically significant differences were observed among the four groups in the UCAS Japan score parameters, including age, hypertension, location and size of the cerebral aneurysm, and the presence of a daughter sac (Table 1). No significant differences were noted in any variables outside the UCAS Japan score components.

**Table 1:** Patient characteristics and the UCAS Japan score HT: hypertension, DM: diabetes mellitus, A-com: anterior communicating artery, ICPC: internal carotid-posterior communicating artery, MCA: middle cerebral artery, BA: basilar artery, ACA: anterior cerebral artery, VA: vertebral artery, IC: internal carotid artery. **p < 0.01. ***p < 0.001.

UCAS Japan score	I			II			III			IV			p-value	
n	30			54			40			13				
Age	56.2 ± 13.6			60.4 ± 15.5			68.9 ± 14.0			71.5 ± 8.7			<0.001	***
Sex (M:F)	16:14			19:35			12:28			3:10			0.265	
HT (yes, no)	5:25			22:32			24:16			9:4			0.003	**
DM (yes, no)	3:27			2:52			3:37			2:11			0.811	
Dyslipidemia (yes, no)	8:22			14:40			14:26			3:10			0.889	
Smoking (yes, no)	15:15			28:26			19:21			5:8			0.961	
Drinking (yes, no)	18:12			32:22			21:19			6:7			0.928	
Anti-platelet agents and/or anticoagulants	2:28			4:50			5:35			1:12			0.954	
Aneurysm location														
A-com	1			18			17			2			<0.001	***
ICPC	0			14			16			6				
MCA	9			12			7			3				
BA	1			1			0			0				
ACA	5			7			0			0				
VA	7			2			0			1				
IC not ICPC	7			0			0			1				
Aneurysm size (mm)														
Major axis	3.9 ± 1.6			4.7 ± 2.4			6.0 ± 2.1			15.7 ± 10.6			<0.001	***
Width	3.5 ± 1.5			4.4 ± 2.4			5.2 ± 2.2			11.5 ± 7.4			<0.001	***
Depth	2.3 ± 1.2			4.6 ± 2.4			5.2 ± 2.2			12.0 ± 10.3			<0.001	***
Neck	2.3 ± 1.2			2.9 ± 2.1			3.1 ± 1.4			5.2 ± 2.5			<0.001	***
Aneurysm volume (mL)	0.26 ± 0.27			0.81 ± 2.63			0.94 ± 1.00			26.45 ± 7.28			<0.001	***
Dome-to-neck ratio	1.6 ± 0.6			1.6 ± 0.6			1.7 ± 0.6			2.3 ± 1.1			0.052	
Bleb (yes, no)	12:18			31:23			36:4			11:2			<0.001	***
Multiple aneurysms	2:28			12:42			4:36			1:12			0.314	

Weather conditions and the UCAS Japan score

Meteorological variables, including temperature, atmospheric pressure, precipitation, and season, were analyzed across UCAS Japan score Groups I-IV (Table 2).

**Table 2:** Weather conditions and the UCAS Japan score *p < 0.05.

UCAS Japan score	I			II			III			IV			p-value	
															Temperature at onset (℃)														
Average	16.1 ± 7.7			17.1 ± 7.4			18.2 ± 7.2			16.4 ± 7.5			0.663																
Max	19.7 ± 7.6			20.8 ± 7.7			21.6 ± 7.7			19.3 ± 7.8			0.702	
Min	12.7 ± 8.4			13.8 ± 7.6			15.1 ± 7.4			13.5 ± 7.8			0.559	
Difference														
Daily	-7.0 ± 2.5			-7.0 ± 2.3			-6.5 ± 2.2			-5.8 ± 1.8			0.267	
One day ago	-0.3 ± 2.6			0.1 ± 1.9			0.7 ± 1.8			-1.6 ± 2.1			0.017	*
Two days ago	-0.5 ± 3.1			0.3 ± 2.6			0.7 ± 2.3			-2.1 ± 2.7			0.013	*
Three days ago	-0.8 ± 3.2			-0.1 ± 3.5			0.6 ± 3.0			-2.5 ± 2.7			0.032	*
Four days ago	-0.3 ± 4.0			0.1 ± 3.6			0.2 ± 3.6			-1.9 ± 2.7			0.262	
Five days ago	0.0 ± 3.6			0.2 ± 3.7			0.5 ± 3.7			-1.5 ± 2.7			0.394	
Atmospheric pressure at onset (hPa)														
Average	1008.9 ± 7.6			1010.2 ± 7.6			1010.3 ± 8.3			1011.6 ± 5.0			0.760	
Difference														
One day ago	0.2 ± 7.0			-0.5 ± 6.1			-1.8 ± 5.0			2.3 ± 6.2			0.231	
Two days ago	-0.8 ± 10.2			-1.3 ± 9.4			-1.9 ± 9.6			1.6 ± 6.5			0.810	
Three days ago	-1.0 ± 9.1			-1.3 ± 9.5			-0.9 ± 9.8			0.2 ± 5.8			0.963	
Four days ago	-3.5 ± 7.9			-1.8 ± 7.7			-0.7 ± 10.1			-0.8 ± 6.3			0.505	
Five days ago	-3.6 ± 7.7			-1.4 ± 7.7			-1.6 ± 10.8			1.3 ± 7.2			0.239	
Precipitation (mm)	5.7 ± 23.2			5.4 ± 13.1			6.5 ± 20.7			9.1 ± 28.2			0.589	
Season														
Spring	14			19			10			6			0.799	
Summer	6			13			9			3				
Autumn	4			13			15			2				
Winter	6			9			6			2				

Statistically significant differences were observed among the four groups in terms of the mean temperature on the day of onset and the mean temperatures 1, 2, and 3 days before onset (p < 0.05). Furthermore, no significant differences were observed in atmospheric pressure, precipitation, or season. Subsequently, pairwise comparisons were performed for the weather variables that showed significant differences (Table 3).

**Table 3:** Comparison of two groups among UCAS Japan score groups, with differences in 1, 2, and 3 days prior *p < 0.05. **p < 0.01.

UCAS Japan score	p-value					
	One day ago		Two days ago		Three days ago	
I vs II	0.961		0.754		0.848	
I vs III	0.599		0.418		0.526	
I vs IV	0.319		0.230		0.266	
II vs III	0.464		0.899		0.859	
II vs IV	0.074		0.029	*	0.093	
III vs IV	0.004	**	0.007	**	0.012	*
I vs II + III + IV	0.643		0.411		0.505	
I + II vs III + IV	0.667		0.635		0.622	
I + II + III vs IV	0.006	**	0.004	**	0.009	**

The temperature difference between 1 day before onset and the day of onset was significantly greater in Group IV compared to Group II (p < 0.05). Additionally, the temperature differences between the day of onset and 1, 2, and 3 days prior were significantly larger in Group IV than in Group III (p < 0.01, p < 0.01, and p < 0.05, respectively). The temperature differences between the day of onset and 1, 2, and 3 days prior were significantly larger in Group IV than in Groups I, II, and III (p < 0.01).

## Discussion

Numerous studies have reported a higher incidence of SAH on colder days [4,5,17-19]. Notably, not only low absolute temperatures but also abrupt temperature drops from the previous day may serve as triggers for SAH, even during warmer seasons [6,20]. For instance, a 1°C decrease in the monthly average temperature has been associated with a 0.5% increase in SAH incidence [21], while an 8°C drop from the previous day may raise the risk by as much as 35% [3]. Variations in barometric pressure have also been implicated: both decreases [2,8] and sharp increases [4,7] have been linked to higher rates of SAH incidence. Additionally, reduced sunlight exposure and lower relative humidity may contribute to increased risk [9]. However, some studies have found no significant association between weather patterns and SAH incidence [10,11,22,23], with some reviews questioning the relevance of weather as a risk factor, citing its low predictive value [10,23] or limited clinical significance [11,22]. Taken together, these findings highlight the complexity of the relationship between weather conditions and SAH, suggesting that the risk of aneurysmal rupture under varying environmental factors requires further, more nuanced investigation.

In this study, the UCAS Japan score was utilized to investigate the relationship between systemic and local risk factors for cerebral aneurysm rupture and weather conditions. This retrospective analysis, which applied the UCAS Japan score to cases of ruptured cerebral aneurysms, may also contribute to validating the score's utility. Notably, this study is also novel in its comprehensive assessment of the association between aneurysm risk and meteorological factors such as temperature, atmospheric pressure, and humidity. The results revealed that in Group IV (the highest risk category), the temperature on the day of SAH onset was significantly lower than that observed 1, 2, and 3 days prior, compared to Groups I, II, and III. These findings suggest that patients in the highest UCAS Japan risk category may be more susceptible to aneurysmal rupture in response to lower ambient temperatures than those in lower-risk groups.

The UCAS Japan score integrates both systemic factors (e.g., hypertension, age, and sex) and local aneurysmal factors (e.g., size, location, and presence of a daughter sac) [15]. Among these, hypertension is particularly susceptible to environmental temperature fluctuations. Studies have shown that both short- and long-term decreases in ambient temperature can elevate blood pressure by approximately 0.4 mmHg systolic and 0.1-0.3 mmHg diastolic per 1°C drop [24], and by as much as 20-30 mmHg during sudden cold exposure [25]. Seasonal analyses have also shown that blood pressure tends to be higher and more variable in winter [26], with increased mortality reported in elderly hypertensive individuals during periods of temperature instability [27]. However, within the UCAS Japan scoring system, hypertension contributes only one point out of a possible 15, and its influence is limited in patients classified within the highest risk category (score ≥9). Thus, temperature-induced elevations in blood pressure alone are unlikely to fully account for the increased rupture risk observed in high-risk groups. Rather, rupture risk appears more strongly associated with local aneurysmal factors, particularly aneurysm size. The hazard ratio for rupture increases substantially with aneurysm size: 1.13 for aneurysms measuring 5-6 mm, 3.35 for 7-9 mm, 9.09 for 10-24 mm, and 76.26 for >25 mm, compared to aneurysms measuring 3-4 mm [14]. Supporting this, Li et al. also found that ruptured aneurysms in SAH patients were significantly larger than unruptured ones [28]. These findings suggest that Group IV, who present with higher rupture risk due to unfavorable local aneurysmal features, may be more susceptible to SAH when triggered by blood pressure fluctuations induced by temperature drops.

Study limitations

The UCAS Japan score is a risk stratification tool designed for unruptured cerebral aneurysms and does not account for ruptured aneurysms. Ruptured cerebral aneurysms may exhibit altered shapes due to changes in their size or the development of a daughter sac. Moreover, the relatively small sample size in Group IV (n = 13) necessitates a cautious interpretation of the findings. Although this study investigated the presence or absence of hypertension, it did not measure actual blood pressure fluctuations in response to temperature changes. Furthermore, due to its retrospective nature, actual blood pressure measurements were not available, and misclassification of patients as “non-hypertensive” despite a history of hypertension could introduce bias, potentially obscuring causal relationships. Additional limitations include the single-center design and the exclusive use of a Japanese patient population, which may restrict the generalizability of the findings. Furthermore, unmeasured confounding variables, such as medication adherence, comorbidities, level of indoor/outdoor activities, and regional variation, may also have influenced the observed associations between weather conditions and aneurysmal rupture.

## Conclusions

These findings suggest that patients classified as high-risk (UCAS Japan IV) may be more susceptible to developing SAH following significant temperature drops. This study highlights the importance of considering environmental temperature fluctuations when assessing aneurysm rupture risk. Future studies should aim to include real-time blood pressure monitoring and a more geographically diverse population to validate and expand upon these results.
